# A Case Control Study on Risk Factors Associated with Low Birth Weight Babies in Eastern Nepal

**DOI:** 10.1155/2015/807373

**Published:** 2015-12-10

**Authors:** Ravi Kumar Bhaskar, Krishna Kumar Deo, Uttam Neupane, Subhadra Chaudhary Bhaskar, Birendra Kumar Yadav, Hanoon P. Pokharel, Paras Kumar Pokharel

**Affiliations:** ^1^Department of Community Medicine and Public Health, National Medical College, Birgunj, Parsa 44300, Nepal; ^2^HelpAge International, Kathmandu 44600, Nepal; ^3^Research Triangle Institute International, Kathmandu 44600, Nepal; ^4^Narayani Sub-Regional Hospital, Birgunj, Parsa 44300, Nepal; ^5^School of Public Health and Community Medicine, B.P. Koirala Institute of Health Sciences, Dharan, Sunsari 56700, Nepal; ^6^Department of Obstetrics and Gynaecology, B.P. Koirala Institute of Health Sciences, Dharan, Sunsari 56700, Nepal

## Abstract

*Background*. This study was done to assess the maternal and sociodemographic factors associated with low birth weight (LBW) babies.* Methods*. An unmatched case control study was done involving 159 cases (mothers having LBW singleton babies) and 159 controls (mothers having normal birth weight singleton babies).* Results*. More than 50% of LBW babies were from the mothers with height ≤145 cm while only 9.43% of NBW babies were from the mothers with that height. Finally, after multivariate logistic regression analysis, maternal height, time of first antenatal care (ANC) visit, number of ANC visits, iron supplementation, calcium supplementation, maternal education, any illness during pregnancy, and hypertension were found as the significant predictors of LBW. However, maternal blood group AB, normal maternal Body Mass Index (BMI), mother's age of 30 or more years, and starting ANC visit earlier were found to be protective for LBW.* Conclusion*. Study findings suggest that selectively targeted interventions such as delay age at first pregnancy, improving maternal education and nutrition, and iron and calcium supplementation can prevent LBW in Nepal.

## 1. Introduction

Low birth weight (LBW) has been defined by WHO as weight at birth of less than 2.5 kg [1]. By international agreement, LBW has been defined as a birth weight of less than 2500 grams, with the measurement being taken preferably within the first hour of life, before significant postnatal weight loss has occurred [2]. It contributes substantially to neonatal, infant, and childhood mortality and morbidity [3].

Across the world, neonatal mortality is 20 times more likely for LBW babies compared to NBW babies (>2.5 kg) [4]. It is now a well recognized fact that birth weight is not only a critical determinant of child survival, growth, and development, but also a valuable indicator of maternal health, nutrition, and quality of life [5].

The incidence of LBW is estimated to be 16% worldwide, 19% in the least developed and developing countries, and 7% in the developed countries. The incidence of LBW is 31% in South Asia followed by East and North Africa (15%), Sub-Saharan Africa (14%), and East Asia and Pacific (7%). Asia accounts for 75% of worldwide LBW followed by Africa (20%) and Latin America (5%). In Nepal, the LBW prevalence is relatively high, ranging from 14 to 32%, as documented from various hospital and community based studies [6]. In a study done in Nepal, the LBW rate was found to be 27% out of which LBW babies at term constitute 70% and LBW babies before term (preterm) constitute 30% [7].

Only 36% of children born in Nepal were weighed at birth. Among them, 12% are of low birth weight. The percentage of children with LBW varies from 15% in mountains to 13% in* Pahad* (hills) and 12% in* Terai* (plain land areas) [8].

This study was done to assess the maternal and sociodemographic factors associated with LBW babies, an important indicator of maternal and newborn health in Nepal.

## 2. Materials and Methods

A hospital based unmatched case control study was carried out at two hospitals: B.P. Koirala Institute of Health Sciences (BPKIHS), Dharan, and Koshi Zonal Hospital (KZH), Biratnagar, in east Nepal. Both hospitals are referral hospitals in the region. BPKIHS is 700 bedded teaching hospital while KZH is 300 bedded hospital. The high uptake of services at these hospitals is probably owing to their low cost. After admission, the majority of births take place within 2 days. The hospital stay is usually at least 1 day after delivery unless the mother or infant experiences problems or the mode of delivery was surgical.

The study data were collected between September 2011 and February 2012 by using interview technique. Eligible mothers were interviewed face to face within 24 hours after delivery. Abstraction of ANC cards and medical records of mothers were also made and anthropometric measurements were taken after interview.

### 2.1. Cases and Controls

Mothers delivering live born singleton term baby with birth weight less than 2500 gm were taken as cases, while mothers delivering live born singleton term baby with birth weight 2500 gm or more were taken as controls. The mothers delivering babies of more than 4 kilograms or babies with congenital anomalies or twins or preterm babies were excluded from the study.

### 2.2. Sample Size

Sample size was estimated using software Epi Info 7.0 version and cross-checked using software nMaster 2.0 version. The sample size estimation was done taking 80% power, 5% alpha error, and 2 as anticipated odds ratio. One hundred and fifty-nine cases and the same number of controls were included in the study. Questionnaire was translated into local language and pretested before data collection. Mothers were interviewed by researchers only.

### 2.3. Ethical Issues

Ethical clearance was obtained from the Institutional Ethical Review Board of BPKIHS. Before starting the interview, verbal individual consent for participation was taken after the study's aim, methods, benefits, and the potential discomfort were adequately explained. Subjects were informed that they were free to abstain or to withdraw from participation at any time. Questions were asked in a way that did not hurt their dignity. The respondents were assured that the answers they give would remain private, anonymous, and confidential.

### 2.4. Variables

The questionnaire contained the variables on maternal factors (age, weight, height, BMI, parity, ANC check-up, iron (60 mg daily) and calcium (500 mg) supplementation, and interpregnancy interval), sociodemographic factors (religion, ethnicity, occupation, socioeconomic status, educational status of parents, type of family, geographical area, and sex of baby), and diseases during pregnancy (anaemia, night blindness, hypertension, heart diseases, tuberculosis, and eclampsia).

### 2.5. Statistical Analyses

The filled questionnaires were checked and rechecked for their completeness at the end of the day during data collection. They were coded before the data entry. Data were entered into the MS Excel 2007 version and were analyzed using PASW Statistical software 18.0. Crude and adjusted odds ratios were used to investigate the factors affecting incidence of LBW, by bivariate and multivariate logistic regression, respectively. Bivariate associations between independent variables and low birth weight were analyzed using simple logistic regression, and crude odds ratios and confidence intervals were calculated. Chi-square analysis was used to test possible bivariate associations between independent variables and low birth weight.

Based on the bivariate analysis and a priori information, a multivariate logistic regression model was constructed to examine the relationship between variables and low birth weight, while also considering possible covariate effects. Entry of the exposure variable was forced, followed by stepwise entry of potentially relevant covariates. The Hosmer-Lemeshow test was used to identify and exclude variables that caused poor fit in the model. Independent associations between variables were characterized by adjusted odds ratios and 95% confidence intervals.

## 3. Results

A total sample of 318 mothers comprising 159 cases and 159 controls were included in the study. Out of 318 subjects, 70.75 percent were from rural area while 29.25 percent were from urban area. More than seventy percent of both the case (70.44%) and the control (71.07%) groups were from rural area. Majority of the subjects were Hindu (94.02%). The rest were Muslims (5.03%) and Christians (0.95%). Majority of the cases (92.45%) and controls (95.60) were Hindus. Although they were very few in number, all Hill occupational castes (ethnic groups from hilly area of the country who economically depend upon their traditional occupation) were cases. Muslims were three times more in percentage in case group (7.55%) than control (2.51%) group while Hill natives were three times more in the control (23.27%) group than in the case (7.55%) group.

Only 24.85% of total respondents belonged to nuclear family, while the rest belonged to joint (64.46%) and extended family (10.69%). Only 10.03% of the total controls were illiterate, while 24.53% of the cases were illiterate.

More than three-fourths of the mothers were housewives while the rest worked outside home. Only 3.14% of the mothers were involved in business while 7.55% of them were laborers.

Majority (46.86%) of the respondent mothers were of 20–24 years. Teenage mothers constituted 19.18% of total mothers. More than one-fifth (22.01%) of the cases were born by teenage mothers, while teenage mothers gave birth to 16.36% of the controls.

Nearly two-thirds (63.52%) of the respondents were below poverty level. Based on modified Kuppuswamy's socioeconomic scale, 44.34% of total mothers were from the upper lower class while 4.41% of them were also from lower class. All the mothers from the lower class gave birth to cases.

There was no significant difference in the maternal age between cases and controls. However, the mothers of LBW infants had significantly lower weight than the controls (*p* < 0.05).  Table 1 compares the basic variables between case and control groups.

Table 2 explains about the maternal risk factors associated with low birth weight babies. Maternal age, maternal weight, maternal height, and interpregnancy interval were found to be associated with low birth weight (Table 2).

The chances of delivering LBW babies were found to be increased as the mothers started the first ANC visit lately. The association between LBW neonates and the time of the first ANC visit was found to be significantly associated (*p* < 0.05). The mothers who had their first ANC in the third trimester of pregnancy were threefold (OR = 3.34, 95% CI: 1.14–9.78) more likely to give birth to LBW neonates than the mothers who had their first ANC in the first trimester. The chance of having LBW babies was also significantly higher in the mothers who had their first ANC in second trimester (OR = 1.65, 95% CI: 1.03–2.63).

LBW was found to be significantly associated with the total number of ANC visits (*p* < 0.001). The mothers who had 1 to 2 ANC visits were 16-fold (OR = 16.74, 95% CI: 6.71–41.95) more prone to have LBW neonates than the mothers who had more than 4 ANC visits in total. The chance of delivering LBW neonates by the mothers who had total ANC visits of 3-4 times was also higher (OR = 3.03, 95% CI: 1.77–5.21).

Iron supplementation was found to be significantly associated with LBW (*p* < 0.001). The mothers who were supplemented with iron for 90 or fewer days were nearly threefold more prone to have LBW babies than the mothers having iron supplementation for more than 90 days.

More than half of the cases (51.57%) had not taken calcium supplement, while less than one-third of the controls (30.19%) had not taken the calcium supplement. In control group, the proportion of having calcium supplement for 90 days or more was found more than in the case group. It was more than double the proportion in the controls (40.25%) compared to the cases (18.24%). The mothers having no calcium supplement were more likely to have LBW babies than mothers having it for 90 days or more (OR = 3.77, 95% CI: 2.14–6.63). The mothers having iron supplement for 90 or fewer days were also more prone to deliver LBW babies (OR = 3.21, 95% CI: 1.72–5.99).

The association between maternal blood group and LBW babies was also found to be significantly associated (*p* < 0.05). Maternal blood group AB has some protective effect against delivering low birth weight babies (OR = 0.33, 95% CI: 0.13–0.80).

Maternal residence (*p* = 0.902), religion (*p* = 0.236), paternal education (*p* = 0.213), paternal occupation (*p* = 0.251), socioeconomic status based on* modified Kuppuswamy scale* (*p* = 0.48), and per-capita income (*p* = 0.162) were found to be insignificant. But caste (*p* < 0.001), type of family (*p* = 0.013), maternal education (*p* = 0.001), maternal occupation (*p* = 0.037), and type of house (*p* = 0.009) were found to be significantly associated with LBW. The odds ratio was found significant for Muslims (OR = 4.69, 95% CI: 1.28–17.10) and major Hill castes (OR = 2.37, CI: 1.08–5.19). Illiterate mothers were more at risk to have LBW babies than mothers who were educated up to SLC or more (OR = 3.04, 95% CI: 1.54–5.98). Laborer mothers were three times more common to deliver LBW babies than housewives (OR = 3.22, 95% CI: 1.24–8.39). Mothers having katcha (house made of bamboo or mud or other local materials) house were twofold more likely to have LBW neonates than mothers having pucca houses (houses made of bricks, rod, and cement) (OR = 2.06, 95% CI: 1.19–3.55).

Any illness during pregnancy was significantly associated with LBW (*p* < 0.05). Mothers having any illness during pregnancy were at more than twofold risk to have LBW babies compared to mothers having no illness (OR = 2.49, 95% CI: 1.46–4.25). Mothers having fever during pregnancy, night blindness, and pain in abdomen during pregnancy were not significantly associated with LBW babies. But hypertension was found to be significantly associated with low birth weight babies. Mothers having hypertension during pregnancy were four times more likely to deliver LBW neonates than mothers having no hypertension (OR = 4.25, 95% CI: 1.17–15.35). Anaemic mothers were also more likely to deliver LBW babies (OR = 1.65; 95% CI: 1.05–2.59).

Exposure variables, maternal weight, height, age, time of first ANC visit, total ANC visits, iron supplementation, calcium supplementation, maternal blood group, maternal education, any illness during pregnancy, hypertension, anaemia, BMI, poverty level (per-capita income), and house type, were included in the multivariate regression analysis. Maternal height, time of first ANC visit, total number of ANC visits, iron supplementation, calcium supplementation, maternal education, any illness during pregnancy, and hypertension are the significant predictors of LBW (Table 3).

Maternal blood group AB, normal maternal BMI, mother's age of 30 years or more, and starting ANC visit earlier were found to be significantly protective for LBW from multivariate regression analysis. Likewise, maternal blood group AB and normal maternal BMI had also protective effect on LBW after adjustment for other variables (Table 3).

The variables maternal weight, iron supplementation, anaemia, and poverty level were found to be significantly associated with LBW during univariate logistic analysis; however, those were found to be insignificant after being adjusted for other variables. It means odds ratios were significant, but adjusted odds ratios were not significant.

## 4. Discussion

In this study, maternal age had no significant association with LBW which is consistent with studies conducted by Mavalankar et al. [9] in India and Fikree and Berenes [10] in Pakistan. But, in contrast, Yadav et al. [11] and Joshi et al. [12] found more risk of delivering LBW babies by teenage mothers.

Maternal postpartum weight in this study was significantly associated (*p* < 0.001) with the birth weight of the baby which is consistent with some studies [9, 13]. Low birth weight babies were found to be nearly 5 times (OR = 4.91, 95% CI: 2.64–9.11) more common in the mothers having 45 kilograms or less weight which is in accordance with the study in Nepal [14].

Reliable information on prepregnancy weight or weight gain during pregnancy could not be obtained due to case control nature of this study, and postpartum weight was used. Mollar et al. have shown in African women with a total pregnancy weight gain of 6 kg that maternal weight within 24 hours postpartum was equal to weight at 14 weeks of gestation [15]. As mean weight gain during pregnancy in India was only about 6 kg in a study by Anderson [16], it is felt that postpartum weight closely reflects prepregnancy weight in our population. Therefore, postpartum BMI closely reflects prepregnancy BMI.

The association between maternal height and LBW was significantly associated in this study as found in other studies [17], but it is contrasted with another study [14].

The relationship between a low maternal BMI and LBW has been known for several decades. Some studies [9, 18] also had the same findings that low maternal BMI was significantly associated with LBW. But, in contrast, low maternal BMI (<18.5 kg/m^2^) was not found to be significantly associated with LBW babies in this study which is in accordance with the study by Ojha and Malla [14].

Interpregnancy interval was found to be significantly associated with LBW babies (*p* < 0.05) in this study which is in agreement with Roy et al. [5] and Mumbare et al. [19] but is in contrast with Yadav et al. [20].

The time of the first ANC visit was found to be significantly associated with LBW in this study and it was in accordance with the studies by Kercher [21] but in contrast with Yadav et al. [20].

Singh et al. [17] support the finding of this study that LBW was found to be significantly associated with the total number of ANC visits.

Intake of iron supplements during pregnancy was found to have a protective effect with respect to LBW in this study which is consistent with Rizvi et al. [3]. Maternal anemia in this study was found to be associated with low birth weight when cutoff was Hb < 12 gm/dL, which is in accordance with the studies by Rizvi et al. [3]. Hypertension was found to be significantly associated with low birth weight babies in this study. The same was documented by Aghamolaei et al. [22]. Aghamolaei et al. [22] also support our study that bleeding during pregnancy was found to be significantly associated with LBW babies.

Maternal education was found to be significantly associated with LBW babies in this study as in some previous studies [23], but it is in contrast with Aghamolaei et al. [22]. This study shows insignificant association between birth weight and religion which was suggested by Yadav et al. [20].

Maternal occupation was found to be significantly associated with LBW babies in our study which is in accordance with some studies [22, 23] but in divergence with Yadav et al. [20]. Per-capita income was not found to be associated with LBW babies in this study which was supported by Yadav et al. [20] but denied by Joshi et al. [12].

LBW babies were more common in mothers having katcha houses than mothers having pucca houses in our study. Rizvi et al. [3] suggest the same finding, while Yadav et al. [20] deny it. As katcha houses reflect poverty in Nepalese society, our finding was also supported by Khan and Jamal's study [24] where they found poverty as a significant factor for LBW.

There was significant negative association between LBW and maternal blood group AB in our study which is in accordance with the findings of Fedrick and Adelstein [25].

Exposure variables, maternal weight, height, age, time of first ANC visit, total ANC visits, iron supplementation, calcium supplementation, maternal blood group, maternal education, any illness during pregnancy, hypertension, anaemia, BMI, poverty level (per-capita income), and house type, were included in the multivariate regression analysis.

Out of those exposure variables, maternal height, time of first ANC visit, total number of ANC visits, iron supplementation, calcium supplementation, maternal education, any illness during pregnancy, and hypertension were found to be the significant predictors of LBW.

Maternal blood group AB, normal maternal BMI, mother's age of 30 years or more, and starting ANC visit earlier were some variables which were found to be significant but with protective effect for LBW.

## 5. Conclusion

Study findings suggest that selectively targeted interventions such as delay age at first pregnancy, improving maternal education and nutrition, and iron and calcium supplementation can prevent LBW in Nepal.

It is suggested to conduct more comprehensive studies to assess maternal blood groups and different diseases during pregnancy as associated factors for low birth weight babies.

## Figures and Tables

**Table 1:** Comparison of basic variables of mothers between cases and controls.

Characteristics	* *Case group		* *Control group		*p* value
	Mean	Std. deviation	Mean	Std. deviation	
Birth weight (gm)	2126.73	340.13	3083.65	332.26	<0.001^*∗*^
Maternal age (years)	24.07	5.09	23.38	5.01	0.23
Family size (number)	5.58	2.51	5.45	2.39	0.63
Monthly income (Rs.)	12627.17	8486.16	16332.70	13786.85	0.003^@^
Maternal Weight (kg)	47.04	9.30	52.11	8.58	<0.001^*∗*^
Total ANC visits (number)	3.36	1.47	4.46	1.44	<0.001^*∗*^
Iron tablet (number)	98.30	47.40	129.81	48.11	<0.001^*∗*^
Calcium tablet (number)	40.94	54.12	72.64	72.81	<0.001^@^
Hb level (gm/dL)	11.33	1.09	11.33	1.22	0.02^*∗*^
Maternal height (cm)	146.73	7.88	152.77	6.04	<0.001^*∗*^
Per-capita income ($)	1.10	0.72	1.39	1.09	0.004^@^

^*∗*^Significant *t*-test at *p* < 0.05, ^@^Mann-Whitney *U* test significant at *p* < 0.05.

**Table 2:** Maternal risk factors associated with low birth weight babies.

Characteristics	* *Case		* *Controls		OR (95% CI)	*p* value
	*N* = 159	Percentage	*N* = 159	Percentage		
Maternal age (years)						
<20	35	22.01	26	16.36	1.52 (0.86–2.71)	>0.05
20–29	98	61.63	111	69.81	1	
≥30	26	16.36	22	13.83	1.34 (0.71–2.51)	
Maternal weight						
≤45 kg	80	50.31	39	24.50	**4.91 (2.64–9.11)**	<0.001^*∗*^
45–55 kg	56	35.23	65	40.9	**2.06 (1.13–3.77)**	
≥55 kg	23	14.46	55	34.60	1	
Maternal height						
≤1.45 meters	85	53.50	15	9.43	**11.09 (5.27–23.33)**	<0.001^*∗*^
1.46–1.55 meters	51	32.08	99	62.27	1.01 (0.55–1.85)	
>1.55 meters	23	14.46	45	28.30	1	
BMI						
<18.5	26	16.35	14	8.80	0.93 (0.53–1.63)	0.12
18.5–24.99	102	64.15	113	71.07	1.92 (0.85–4.34)	
≥25	31	19.50	32	20.13	1	
Parity						
1	93	58.49	98	61.63	0.97 (0.60–1.56)	0.41
2	52	32.71	53	33.33	1	
3 or more	14	8.80	8	5.03	1.78 (0.69–4.61)	
Interpregnancy interval						
<24 months	23	29.87	14	18.18	**2.65 (1.16–6.06)**	0.03^*∗*^
24–48 months	26	33.76	42	54.55	1	
>48 months	28	36.36	21	27.27	**2.15 (1.02–4.55)**	
Total	77		77			
Socioeconomic status						
Upper lower^@^	78	44.65	70	44.03	1.50 (0.78–2.92)	0.48
Lower middle	61	38.36	62	38.99	1.33 (0.67–2.62)	
Upper middle	20	12.58	27	16.98	1	

^*∗*^Significant (*p* < 0.05); ^@^lower class also included; OR in bold denotes being significant.

**Table 3:** Multivariate regression analysis for possible predictors of LBW.

Characteristics	AOR	AOR at 95% CI	*p* value
Maternal weight			
≤45 kg	1.81	0.36–9.15	0.473
45–55 kg	2.14	0.63–7.26	0.224
≥55 kg	Ref.		
Maternal height			
≤1.45 meters	**20.38**	**4.87–85.22**	**<**0.001^**∗**^
1.46–1.55 meters	1.81	0.64–5.12	0.262
>1.55 meters	Ref.		
Time of 1st ANC visit			
First trimester	**0.03**	**0.002–0.51**	0.015^**∗**^
Second trimester	0.99	0.44–2.25	0.986
Third trimester	Ref.		
ANC visit (number)			
1-2	**172.79**	**23.57–1266.67**	0.001^**∗**^
3-4	**5.93**	**2.26–15.59**	0.001^**∗**^
>4	Ref.		
Iron supplementation			
1–90 days	0.58	0.23–1.49	0.256
91–180 days	Ref.		
Calcium supplementation			
No	1.56	0.57–4.29	0.388
1–90 days	**3.57**	**1.22–10.42**	0.02^**∗**^
91–180 days	Ref.		
Blood group			
A	1.18	0.48–2.92	0.716
AB	**0.17**	**0.04–0.76**	0.021^**∗**^
B	Ref.		
O	1.29	0.51–3.33	0.590
Maternal education			
Illiterate	**5.69**	**1.83–17.68**	0.003^**∗**^
Literate	Ref.		
Any illness during pregnancy			
Yes	**4.24**	**1.90–9.44**	**<**0.001^**∗**^
No	Ref.		
Hypertension			
Yes	**19.44**	**3.11–121.52**	0.002^**∗**^
No	Ref.		
Anaemia			
Yes (Hb < 12 gm/dL)	1.27	0.59–2.71	0.534
No (Hb ≥ 12 gm/dL)	Ref.		
Maternal age			
<20	0.36	0.12–1.08	0.069
20–29	Ref.		
≥30	**0.15**	**0.04–0.53**	0.003^**∗**^
BMI			
Thin (<18.5 kg/m^2^)	0.91	0.13–6.42	0.928
Normal (18.5–24.99 kg/m^2^)	**0.19**	**0.05–0.70**	0.013^**∗**^
Overweight (≥25 kg/m^2^)	Ref.		
Per-capita income			
<$1.25 per day	1.17	0.54–2.57	0.691
≥$1.25 per day	Ref.		
House type			
Katcha	**2.89**	**1.12–7.47**	0.0029^**∗**^
Pucca	Ref.		

^*∗*^Significant (*p* < 0.05); AOR in bold denotes being significant.
